# Supplementary material for: SyNDI: synchronous network data integration framework
Source: BMC Bioinformatics. 2018 Nov 6;19:403. doi: 10.1186/s12859-018-2426-5 (PMC6219086; doi:10.1186/s12859-018-2426-5)
Supplement: Supplementary file 5 — Files of the example presented in Additional file 4. This additional file is a zip package that contains all files used in Additional file 4. Detailed descriptions of each of these files are presented in a README file included in this zip package. (GZ 30411 kb) [file 12859_2018_2426_MOESM5_ESM.gz › SyNDITools/Meme2Fimo/meme_4.9.0_4.tar.gz!/meme_4.9.0/doc/beeml2meme.html]

beeml2meme


# beeml2meme

**Usage:** `beeml2meme [options] <matrix file>`

**Description:**

Convert an BEEML matrix file into a MEME version 4 formatted file
suitable for use with MAST and other MEME Suite programs.

**Input:**

An BEEML matrix file describes one ore more motifs in terms of letter
energy values.

A probability matrix and optionally a log-odds matrix are output for each
motif in the file. The probability matrix is computed using
pseudo-counts consisting of the background frequency (see -bg, below)
multiplied by the total pseudocounts (see -pseudo, below). The log-odds
matrix uses the background frequencies in the denominator and is log
base 2.

**Options:**

-bg <background file>
:   file with background frequencies of letters; default: uniform background

-pseudo <total pseudocounts>
:   add <total pseudocounts> times letter background to each frequency; default: 0

-logodds
:   print log-odds matrix, too; default: print frequency matrix only

-url <website>
:   website for the motif; The BEEML ID is substituted for MOTIF\_NAME; default: no url

**Output:**

Writes MEME format to standard output.

**Sample Input:**

```
# cell08_Alx3_3418, probe_r_sqr = 0.676, 8mer_r_sqr = 0.868
A       0.12    1.48    0       0       2.18    1.15    0       0.09    0.14    0.13
C       0.02    1.69    1.57    2.2     1.7     0.99    0.82    0.36    0       0.27
G       0.42    2.31    1.9     2.8     2.3     1.12    0.16    0       0.25    0.27
T       0       0       1.67    1.62    0       0       0.59    0.52    0.04    0
# cell08_Alx4_1744, probe_r_sqr = 0.756, 8mer_r_sqr = 0.895
A       0       0.73    0.97    0       0       3.35    2.65    0       0       0.25
C       0.36    0       0       1.58    2.82    3.82    2.23    3.44    0.67    0
G       0.16    0.5     1.32    1.86    2.94    6.2     2.42    3.01    0.21    0.11
T       0.25    0.15    0.05    1.59    3.23    0       0       1.98    0.31    0.28
```
